# Leonardite-derived humic substances enhance phosphorus use efficiency in lettuce through integrated physiological and metabolic reprogramming

**DOI:** 10.3389/fpls.2026.1809888

**Published:** 2026-05-28

**Authors:** Santiago Atero-Calvo, Francesco Magro, Giacomo Masetti, Silvia Celletti, Andrea Ertani, Giuseppe Mannino, Cinzia M. Bertea, Juan M. Ruiz, Michela Schiavon

**Affiliations:** 1 Department of Plant Physiology, University of Granada, Granada, Spain; 2 Sofbey S.A., Mendrisio, Switzerland; 3 Department of Agricultural, Forest and Food Sciences (DISAFA), University of Turin, Grugliasco, Turin, Italy; 4 Department of Life Sciences and Systems Biology, University of Turin, Turin, Italy

**Keywords:** antioxidants, biostimulant, phenolic compounds, phosphorus, root morphology

## Abstract

Phosphorus (P) is essential for plant growth, and its deficiency significantly limits crop productivity. Plant biostimulants offer a sustainable approach to enhance plant tolerance to nutrient stress and reduce fertilizer use. Among these, humic substances (HS) derived from leonardite show promise, though their physiological effects under P deficiency remain unclear. This study evaluated the impact of a leonardite-derived HS on lettuce (*Lactuca sativa* L.) grown under two P regimes: high P (HP, 1 mM) and low P (LP, 0.2 mM). HS were applied at two concentrations via root (R1: 0.40; R2: 0.60 mL L^-^¹) or foliar (F1: 7.50; F2: 10.00 mL L^-^¹) treatments. Phosphorus shortage reduced shoot biomass and chlorophyll content, but stimulated root growth, total phenolic content (TPC), and antioxidant capacity. HS improved shoot growth across both P levels, with R2 being most effective at HP. Under LP, both HS doses produced similar effects. The enhanced tolerance to P deficiency conferred by HS was associated with improved root biometric traits, enhanced P acquisition (PAE), P utilization (PUtE), and overall P use efficiency (PUE), along with elevated acid phosphatase activity, chlorophyll, glycine betaine, TPC, and antioxidant capacity. All HS treatments enhanced root elongation; notably, F2 treatment increased root volume and diameter under LP. Furthermore, HS application modulated the leaf phenolic profile: F2 enhanced the accumulation of specific phenolics, while root-applied HS increased TPC and antioxidant response under LP. In conclusion, this leonardite-derived biostimulant demonstrated potential to enhance plant performance under P-limited conditions, supporting its use for more sustainable nutrient management.

## 1 Introduction

Since the green revolution, the external application of phosphorus (P) fertilizers has been widely adopted to increase crop productivity, significantly boosting agricultural output over the years (Bindraban et al., 2020). However, this practice relies heavily on phosphate rock, a non-renewable resource that is being steadily depleted (Shukla and Prithiviraj, 2021). Compounding the issue, only 20-25% of applied P is taken up by crops, while the remaining P becomes fixed by soil minerals, contributes to soil degradation, or is lost through erosion and runoff, potentially leading to eutrophication (Bindraban et al., 2020; Micha et al., 2023). Therefore, developing sustainable strategies to enhance plant P bioavailability, uptake, and use efficiency is a key research priority aimed at reducing reliance on chemical P fertilizers while mitigating their negative environmental impacts (Mirriam et al., 2022; Kumar et al., 2024).

Phosphorus deficiency affects nearly 40% of agricultural soils globally, impacting an estimated 5.7 billion ha of farmland (Roch et al., 2019; Rafiullah et al., 2020). Under such nutritional imbalance, plants undergo various metabolic and physiological changes, including increased levels of Reactive Oxygen Species (ROS), damage to chloroplast structure, and reductions in photosynthesis performance (Shi et al., 2020; Liu et al., 2024). Plants absorb P primarily as inorganic orthophosphate (Pi), which has a low diffusion rate and is taken up more slowly compared to other ions, such as nitrate (NO_3_^−^) (Khan et al., 2023). Although P is abundant in soil, it often becomes immobilized through the formation of complexes with elements such as calcium (Ca) and magnesium (Mg) in calcareous and neutral soils, or with aluminum (Al) and iron (Fe) in acidic soils (Wang et al., 2023a; Fang et al., 2024). In addition, P can be strongly adsorbed onto the surfaces of Fe and Al (hydro)oxides (Santoro et al., 2024). These processes make P the least mobile and bioavailable macronutrient for plants, resulting in P deficiency being one of the most common nutritional stresses that significantly reduce crop productivity (Santoro et al., 2023).

As a response to limited P availability, plants up-regulate the expression and activity of root Pi transporters to enhance P uptake and increase the exudation of organic acids, amino acids, sugars, and protons in the rhizosphere to promote P mobilization. Similarly, the root secretion of acid phosphatase enzymes into the rhizosphere facilitates the release of Pi from organic P compounds (Zhou et al., 2023; Sieczko et al., 2024). Additionally, under low-P conditions, plants increase the biosynthesis of internal phosphatases to improve P use efficiency, along with the accumulation of secondary metabolites, such as phenolic acids and flavonoids, which support antioxidant defense mechanisms (Kaur et al., 2022; Wang et al., 2023b). The enhanced production of compatible solutes, like glycine betaine (GB), may also contribute to maintaining P homeostasis under P-deficient conditions (Li et al., 2019). In terms of root architecture, plants typically stimulate the development and increased density of lateral roots and root hairs, thereby improving P foraging (Zheng et al., 2024).

In this context, the use of plant biostimulants is regarded as an eco-friendly approach to enhance nutritional traits by improving nutrient uptake, assimilation, use efficiency, as well as by increasing crop tolerance to abiotic stresses, thereby supporting plant growth under limiting conditions (Navarro-Morillo et al., 2024; Velasco-Clares et al., 2024). Biostimulants are classified into microbial (plant growth-promoting bacteria and arbuscular mycorrhizal fungi) and non-microbial (protein hydrolysates, amino acids, seaweed and plant extracts, chitosan, and humic substances) categories (Hernandez et al., 2024).

To date, humic substances (HS) are among the most widely marketed biostimulants and can constitute up to 80% of soil organic matter. They consist of three main fractions that can be separated based on their solubility: humin (the insoluble fraction), humic acids (HA, soluble at alkaline pH), and fulvic acids (FA, soluble at both alkaline and acidic pH) (Nardi et al., 2021; Ore et al., 2023). Chemically, HS consist of diverse small organic molecules, including polypeptides, sugars, aliphatic chains, aromatic rings, and fatty acids, assembled through hydrophobic interactions and hydrogen bonds (Piccolo, 2002). The positive impact of HS on plant growth and performance arises from both indirect and direct mechanisms. Indirectly, HS improve soil physico-chemical properties, such as porosity, water-holding capacity, and cation exchange capacity, thereby improving the rhizosphere environment and nutrient availability (Yang et al., 2021; Ore et al., 2023). Direct effects, meanwhile, are associated with the stimulation of primary and secondary metabolic pathways, including nutrient assimilation, photosynthesis capacity, hormone regulation, biosynthesis of antioxidant compounds, among others (da Silva et al., 2021; Atero-Calvo et al., 2024c). The potential role of HS as biostimulants in promoting plant growth under nutrient-limited conditions has been attributed to their ability to form complexes with mineral elements such as iron (Fe), zinc (Zn) or P. Consequently, these complexes enhance the bioavailability of these nutrients and their uptake by plants (Zanin et al., 2019; da Silva et al., 2021). The changes in soil pH induced by HS may also influence P availability and acquisition (Akimbekov et al., 2020). Additionally, HS may modulate the expression of genes involved in nutrient acquisition and use efficiency (Nardi et al., 2017; Santoro et al., 2023). Recent studies have demonstrated the impact of HS on P acquisition. For instance, Piri et al. (2023) reported that leonardite-derived HS increased P release in calcareous soils, thereby improving the growth of Moldavian balm. Similarly, Rosolem et al. (2024) found that leonardite enhanced P diffusion in the soil and P acquisition by maize plants. However, the physiological effects of HS on plant performance depend on various factors, including their source, extraction method, structure, chemical composition, molecular weight, plant species, and rate and method of application (root or foliar) (Jindo et al., 2020; Santoro et al., 2023).

Despite the increasing evidence demonstrating the positive effects of HS on P nutrition, their specific role in modulating physiological responses under low-P conditions remains not fully understood. In particular, limited information is available on (i) the comparative effectiveness of root versus foliar HS application under P deficiency, and (ii) the extent to which HS influence secondary metabolism, especially phenolic compound biosynthesis, as a function of the P nutritional status. Furthermore, most previous studies have primarily focused on plant growth responses or P uptake, while integrated analyses combining physiological, biochemical, and metabolomic responses remain scarce.

Therefore, this work aims to provide an integrated assessment of HS effects on lettuce plants grown under both P-sufficient and P-deficient conditions, combining analyses of P acquisition and use efficiency with morphological, physiological, and metabolomic responses. In addition, the study compares root and foliar application strategies to elucidate their differential impacts on plant performance.

We hypothesized that the applications of leonardite-derived HS could support plant growth under P deficiency by regulating P acquisition and use efficiency processes, as well as modulating the biosynthesis of specific secondary metabolites, particularly phenolic compounds. Consequently, HS-based biostimulant could be a suitable option for reducing the overuse of chemical fertilizers, thereby contributing to more sustainable agriculture. The effectiveness of these HS was assayed in lettuce (*Lactuca sativa* L. var. capitata) plants grown under limited P availability and treated *via* either root or foliar application. Lettuce is an important crop due to its high economic value, short growth cycle, and sensitivity to nutrient and environmental conditions. These traits make this species ideal for intensive production systems, including soil-less cultivation methods. Moreover, its well-defined response to external nutritional inputs makes it a suitable model for evaluating the effects of plant biostimulants under controlled conditions.

## 2 Materials and methods

### 2.1 Plant growth conditions and experimental design

Seeds of *Lactuca sativa* L. var. capitata were soaked in distilled water (dH_2_O) overnight and subsequently surface sterilized using 5% (v/v) sodium hypochlorite for 30 s. Afterwards, seeds were transferred to a tray with cells filled with perlite. Seventeen days after germination (DAG), a total of 120 seedlings were transplanted into individual pots (0.30 L, 1 plant/pot), containing 100% perlite and irrigated with ¼ Hoagland nutrient solution with the following composition: 330 μM NH_4_NO_3_, 1 mM Ca(NO_3_)_2_, 1.6 mM KNO_3_, 500 μM MgSO_4_, 1.7 μM MgCl_2_, 3 μM H_3_BO_3_, 0.6 μM MnSO_4_, 0.05 μM ZnSO_4_, 0.02 μM CuSO_4_, 10 μM FeNaEDTA, and 0.008 μM MoO_3_. The pots were randomly arranged into a growth chamber under controlled environmental conditions with relative humidity of 60%, temperature of 23°C and 12/12h photoperiod at a photosynthetic photon flux density of 250 μmol m^-2^ s^-1^.

At 30 DAG, lettuce plants were divided into two groups depending on the level of P supplied: high-P (HP), which corresponded to an adequate P supply, and low-P (LP), in which plants received 1 mM and 0.2 mM of KH_2_PO_4_, respectively. The reduction in K in the LP group was compensated by adding KCl. Furthermore, within each group, a total of five treatments were carried out based on HS applications: root (R) applications at 0.40 mL L^−1^ (R1) and 0.60 mL L^−1^ (R2), foliar (F) spray at 7.50 mL L^−1^ (F1), and 10.00 mL L^−1^ (F2), and a treatment without HS, which served as the control (C). Three HS applications were carried out at 7-day intervals. R-applications were performed by diluting HS in the previously described nutrient solution and irrigating each plant at the top of the pot (~100 mL); F-applications were carried out by dissolving HS in dH_2_O and spraying ~12.5 mL onto lettuce leaves of each plant; finally, C- and R-applications were performed by spraying lettuce leaves with dH_2_O instead of HS. The source of HS used in this study was a leonardite-derived biostimulant named BLACKJAK^®^, provided by Sofbey S.A. (Mendrisio, Switzerland). Its chemical characterization is detailed in Barone et al. (2019). The doses employed in this experiment were selected based on a previous screening conducted on lettuce grown under sufficient nutrient conditions, in which plant biomass and nutrient accumulation were evaluated (Atero-Calvo et al., 2023; 2024a). The experiment was hence conducted using a completely randomized block design with two P levels and five HS treatments at each level, resulting in a total of ten treatments. Each treatment was replicated with twelve lettuce plants, for a total of 120 plants. The experiment lasted 51 days.

Quality control was ensured throughout the experiment by using a completely randomized design, homogeneous cultivation conditions, and systematic monitoring of environmental parameters. Biological replication (n = 12) was applied for each treatment, and all analyses followed standardized and previously validated protocols. Instrument calibration and the inclusion of technical replicates when applicable were used to ensure data reliability and reproducibility.

### 2.2 Plant harvesting

At the end of the experiment, shoots and roots from each plant were harvested. Roots were gently rinsed with dH_2_O, blotted dry with paper, and weighed to determine their fresh weight (FW). Shoot and root morphological analyses were conducted on five plants per treatment (description in section 2.3.), and their shoots and roots were further oven-dried at 70 °C for 48 h and weighed to determine the dry weight (DW). The aerial parts of the remaining seven plants per treatment were immediately frozen in liquid nitrogen and stored at −20 °C for subsequent biochemical analyses.

### 2.3 Shoot and root morphological measurements

The rosette area of each plant was measured from digital images. Five plants per treatment were used to measure root morphological traits potentially associated with P acquisition, including total root length, surface area, volume, average diameter, and number of tips, using computerized root scanning with WinRHIZO software (Reagent Instruments Inc., Québec, QC, Canada).

### 2.4 Phosphorus quantification and P use efficiency parameters

Phosphorus concentration was determined in oven-dried shoots and roots (50 mg DW) using the green malachite method after sulfuric/perchloric digestion (Ohno and Zibilske, 1991). Shoot P content was estimated by multiplying the P concentration by the shoot DW.

The P Acquisition Efficiency (PAE) was calculated by dividing the P accumulated in shoots (P content) by the P supplied externally (Santoro et al., 2023). Furthermore, P Utilization Efficiency (PUtE) was assayed as the ratio between shoot DW and shoot P content, whereas P Use Efficiency (PUE) was estimated by multiplying PAE by PUtE (Atero-Calvo et al., 2024b).

### 2.5 Determination of shoot acid phosphatase activity

Shoot acid phosphatase activity was determined according to the method described by Hayes et al. (2000), with slight modifications. Briefly, 0.1 g of frozen shoot material was extracted in liquid nitrogen and then homogenized in an extraction buffer containing 15 mM 2-(N-morpholino) ethanesulfonic acid (MES) (pH 5.5), 0.5 mM CaCl_2_·2H_2_O, and 1 mM EDTA. The homogenate was centrifuged at 13,800 × *g* for 15 min at 4 °C, and an aliquot of the supernatant was incubated at 26 °C for 30 min with a reaction buffer consisting of 15 mM MES (pH 5.5), 1 mM EDTA, 5 mM cysteine, and 10 mM *p*-nitrophenyl phosphate (pNPP) as the substrate. The reaction was stopped by adding 0.25 M NaOH, and the concentration of *p*-nitrophenol (pNP) was quantified at 412 nm against a standard curve of pNP, using a Shimadzu UV-1800 spectrophotometer (Shimadzu Corp., Columbia, MD, USA).

The protein concentration in the enzyme extracts was determined using the Bradford assay (Bradford, 1976), with bovine serum albumin (BSA) as the standard.

### 2.6 Determination of chlorophyll content

Chlorophylls (Chl *a* and Chl *b*) were extracted from frozen lettuce leaves using 90% (v/v) methanol. The mixture was then centrifuged at 5,000 ×*g* for 5 min. The absorbance of the resulting supernatant was recorded at three wavelengths: 649 nm, 664 nm, and 470 nm, using a UV-Probe 1280 Spectrophotometer (Shimadzu, Italy). Concentrations of chlorophylls (Chls) were subsequently calculated based on the absorbance readings, following the formulas established by Wellburn (1994):


$$Chl\;a=15.65\times A_{664}-7.34\times A_{649}$$

$$Chl\;b=27.05\times A_{649}-11.21\times A_{664}$$

Total Chls were calculated as the sum of Chl *a* and Chl *b.*

### 2.7 Quantification of glycine betaine content

Glycine betaine was determined from an aqueous extraction of 20 mg of dry leaves during 24 h. After centrifugation at 2,000 ×*g* for 5 min, the supernatant was mixed with 2 N H_2_SO_4_ and incubated at 4 °C for 16 h. Samples were then centrifuged at 9,000 ×*g* for 15 min, and the resulting pellet was resuspended in 1,2-dichloroetane for 2 h. Afterwards, GB was determined at 365 nm against a standard curve using a Tecan spectrophotometer (Tecan Group Ltd, Switzerland) (De la Torre-González et al., 2018).

### 2.8 Total phenolic content and metabolomic analysis

Frozen leaves were extracted using 90% (v/v) methanol, and after centrifugation at 8,000 x *g* for 10 min, the methanolic extracts were employed to determine total phenolic content (TPC) and specific phenolic compounds.

Total phenolic content was measured using the Folin–Ciocalteu reagent. In this assay, phenols are oxidized under alkaline conditions by a yellow molybdotungstophosphoric acid reagent, producing a blue-colored molybdotungstophosphate complex that can be quantified spectrophotometrically at 725 nm (Gatti et al., 2024). Briefly, 20 µL of the sample extract was mixed with 20 µL of Folin reagent, 10 µL of 20% (w/v) sodium carbonate, and distilled water to a final volume of 200 µL. The mixture was then incubated at room temperature for 20 min. Absorbance was measured using a UV-Probe 1280 Spectrophotometer (Shimadzu, Italy). Results were expressed as mg of gallic acid equivalents (GAE) per 100 g of FW.

The phenolic profile was determined using a High-Performance Liquid Chromatography (HPLC) system (Mannino et al., 2022). The analytical system consisted of a LC unit (Agilent Technologies 1200, Santa Clara, CA, USA), coupled to a Diode Array Detector (DAD) and an ion trap mass spectrometer (Agilent Technologies 6300) equipped with an electrospray ionization (ESI) source. Chromatographic separation was performed using a Luna C18 reversed-phase column (3 μm, 150 × 3.0 mm i.d.) maintained at 25 °C *via* a built-in column oven (Agilent 1200). The mobile phase was delivered at a constant flow rate of 0.2 mL min^-1^. UV-Vis spectra of the eluted compounds were recorded across a wavelength range of 220–800 nm. The nitrogen gas flow rate was set at 15.0 mL min^-1^, with the drying gas temperature held at 350 °C. The capillary voltage was adjusted to ±1.5 kV. Identification of compounds was based on Retention Time (RT), UV-Vis spectral characteristics, and Mass Spectrometry (MS) fragmentation patterns, and compared to authentic standards obtained from Sigma-Aldrich (St. Louis, MO, USA). Furthermore, a multistep linear gradient was used, starting at 15% (v/v) solvent B, increasing to 45% over 15 min, and then to 70% by 20 min. After each run, the gradient returned to initial conditions and was held for 10 min before the next injection. The sample injection volume was 10 μL.

### 2.9 Total antioxidant capacity assay

Total antioxidant capacity in lettuce shoots was estimated by the Ferric-Reducing Antioxidant Power (FRAP) assay, based on the reduction of Fe^3+^(ferric ion)-TPTZ (2,4,6-tripyridyl-s-triazine) complex to ferrous form. Frozen shoots were extracted with 90% (v/v) methanol. Afterwards, methanolic extracts were mixed with FRAP reagent composed by 0.3 M acetate buffer (pH 3.6), 10 mM TPTZ, and 20 mM FeCl_3_ in an 8:1:1 (v/v/v) ratio. The mixture was incubated at 37 °C for 30 min with an appropriately diluted sample, and absorbance was recorded at 595 nm employing a UV-Probe 1280 Spectrophotometer (Shimadzu, Italy). Gallic acid served as the standard, and results were expressed as mmol of GAE per mL of biostimulant (Campobenedetto et al., 2021).

### 2.10 Statistical analyses

Data from each treatment (R1, R2, F1, F2, and C) within each P condition (LP or HP) were subjected to a one-way ANOVA analysis at 95% confidence. Means were compared by Fisher’s least significant differences (LSD). Moreover, a *t*-Student test was performed to compare means within each treatment (R1, R2, F1, F2, and C) between both P conditions (LP and HP). The significance levels were expressed as *(P< 0.05), **(P< 0.01), ***(P< 0.001), and NS (not significant, P > 0.05). The Statgraphics Centurion XVI software was employed to perform statistical analysis.

In addition, the phytochemical profiles generated by HPLC-DAD-MS/MS were median-normalized, log_10_-transformed, and scaled using the Pareto method. Metabolomic analyses were conducted using the web-based multi-omics platform MetaboAnalyst 4.0, and differences among samples were visualized using Principal Component Analysis (PCA).

## 3 Results

### 3.1 Shoot and root growth

**Figure 1:**
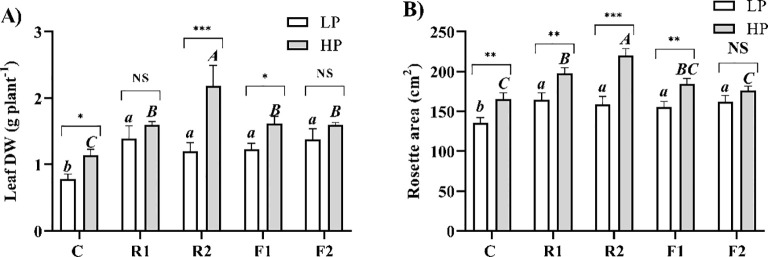
Leaf dry weight (DW) **(A)** and rosette area **(B)** of lettuce plants under low phosphorus (LP) and high phosphorus (HP) and treated with humic substances (HS) applied via root (R) at 0.40 mL L^-1^ (R1) and 0.60 mL L^-1^ (R2), and foliar (F) at 7.50 mL L^-1^ (F1) and 10.00 mL L^-1^ (F2), along with a control (C, HS-untreated). Values are means ± standard errors (n = 12). Different lowercase (LP) and uppercase (HP) letters indicate significant differences among HS treatments within each P level based on one-way ANOVA followed by Fisher’s least significant test (LSD, P< 0.05). Additionally, means values between LP and HP conditions within the same HS treatment were compared using a t-Student test and differences were expressed as *(P< 0.05), **(P< 0.01), ***(P< 0.001), and NS (not significant, P > 0.05).

The supply of low P to lettuce significantly decreased leaf DW compared to HP conditions, except in plants treated with R1 and F2 (**Figure 1A**). Rosette area also reduced under LP across treatments, but in F2-treated plants, it remained unaffected (**Figures 1A, B**). Under HP conditions, the application of the leonardite-derived biostimulant at different doses significantly increased leaf DW compared to untreated plants (+40%, +92%, +42%, and +40% under R1, R2, F1, and F2 treatments, respectively), with the R2 treatment showing the greatest enhancement (+92%) (**Figure 1A**). Similarly, both R1 and R2 treatments significantly increased the rosette area (+19% and +33%, respectively) under HP (**Figure 1B**). Under LP conditions, all HS doses significantly improved leaf DW (+78%, +54%, +58%, and +77%) and rosette area (+22%, +17%, +15%, and +20%), under R1, R2, F1, and F2 treatments, compared to untreated controls. However, no significant differences were observed among the HS doses under LP (**Figures 1A, B**).

**Figure 2:**
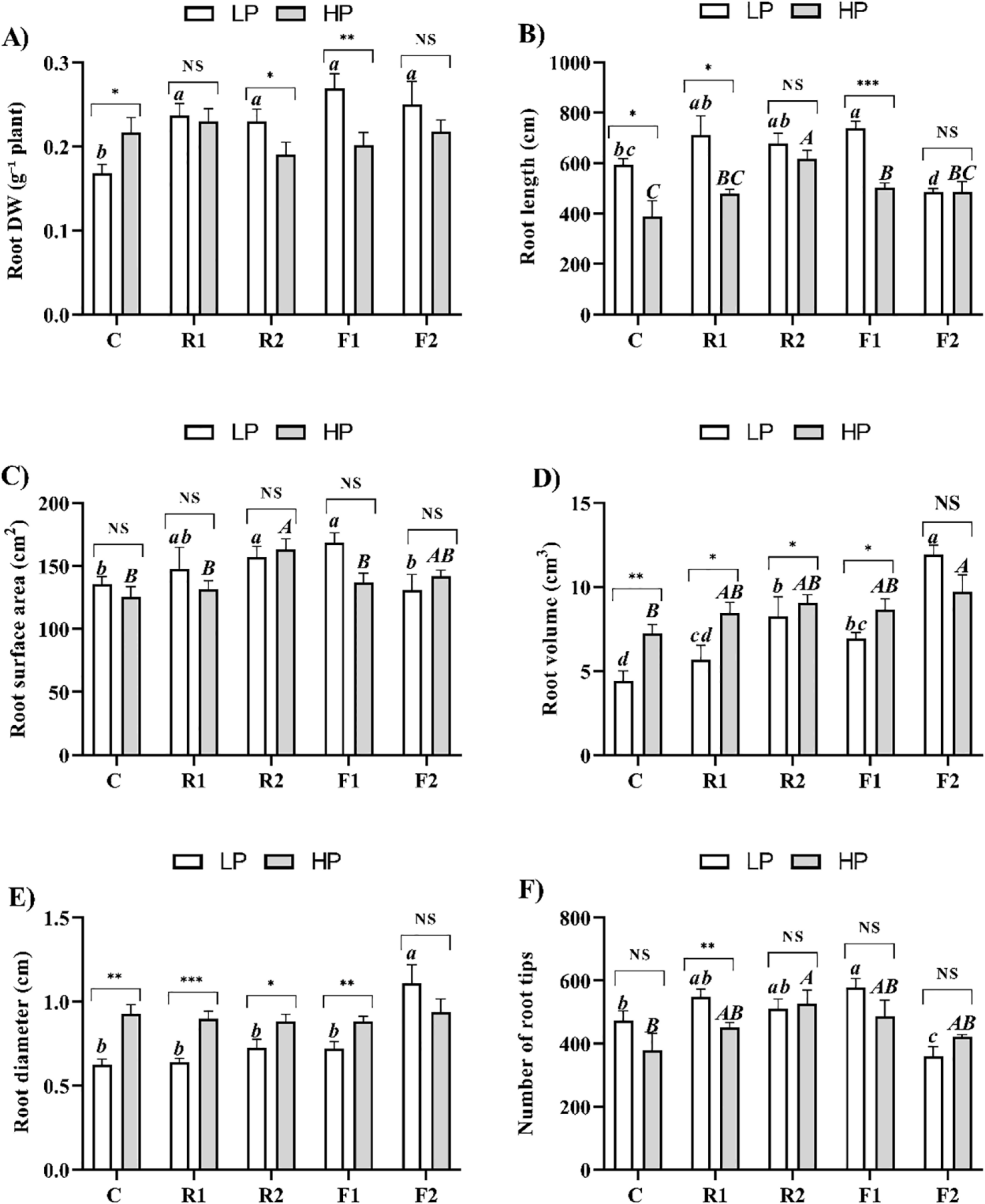
Root dry weight (DW) **(A)**, root length **(B)**, root surface area **(C)**, root volume **(D)**, root diameter **(E)**, and number of root tips **(F)** in lettuce plants under low phosphorus (LP) and high phosphorus (HP) and treated with humic substances (HS) applied via root (R) at 0.40 mL L^-1^ (R1) and 0.60 mL L^-1^ (R2), and foliar (F) at 7.50 mL L^-1^ (F1) and 10.00 mL L^-1^ (F2), along with a control (C, HS-untreated). Values are means ± standard errors (n = 12) Different lowercase (LP) and uppercase (HP) letters indicate significant differences among HS treatments within each P level based on one-way ANOVA followed by Fisher’s least significant test (LSD, P< 0.05). Additionally, means values between LP and HP conditions within the same treatment were compared using a t-student test and differences were expressed as *(P< 0.05), **(P< 0.01), ***(P< 0.001), and NS (not significant, P > 0.05).

Root DW was overall lower in plants grown under LP conditions compared to those grown under HP, except for F2-treated plants, where no significant differences were evident (**Figure 2A**). However, HS treatments to LP plants led to a general increase in root DW (+33%, +35%, +58%, and +47% under R1, R2, F1, and F2 treatments, respectively).

Various biometric parameters related to root growth were also evaluated (**Figures 2A–F**). Phosphorus limitation generally led to an increase in root length (**Figure 2B**) and reductions in both root volume (**Figure 2D**) and root diameter (**Figure 2E**), with these effects being significant for all these traits in HS-untreated plants and those treated with R1 and F1, compared to HP plants. Regarding root diameter, a significant decrease was also observed in plants treated with R2 under LP conditions (**Figure 2E**). In contrast to these root traits, root surface area was not influenced by P supply alone (**Figure 2C**). Similarly, the root tip number did not show significant variation in response to P availability, except in R1-treated plants, which exhibited higher values under LP conditions (**Figure 2F**).

Under HP conditions, the R2 and F1 treatments significantly increased root length by 59% and 30%, respectively (**Figure 2B**). Additionally, R2 alone stimulated the production of root surface area (+30%) (**Figure 2C**) and the number of root tips (+39%) (**Figure 2F**), compared to untreated HP plants. The F2 treatment was instead more effective in enhancing root volume (+34%) (**Figure 2D**).

Under P limitation, the R1 treatment did not alter any of the measured root traits. In contrast, the R2 treatment significantly increased root length (+15%), surface area (+16%) and root volume (+87%) relative to the untreated LP control (**Figures 2B–D**). The F1 treatment elicited the broadest response, raising root length, surface area, volume, and tip number by 24%, 24%, 57% and 22%, respectively (**Figures 2B–D, F**). Conversely, F2 application significantly reduced root length (**Figure 2B**) and tip number (**Figure 2F**), while leading to the highest increase in root volume (+170%) (**Figure 2D**) and root diameter (+83%) (**Figure 2E**).

### 3.2 Phosphorus accumulation and use efficiency

**Figure 3:**
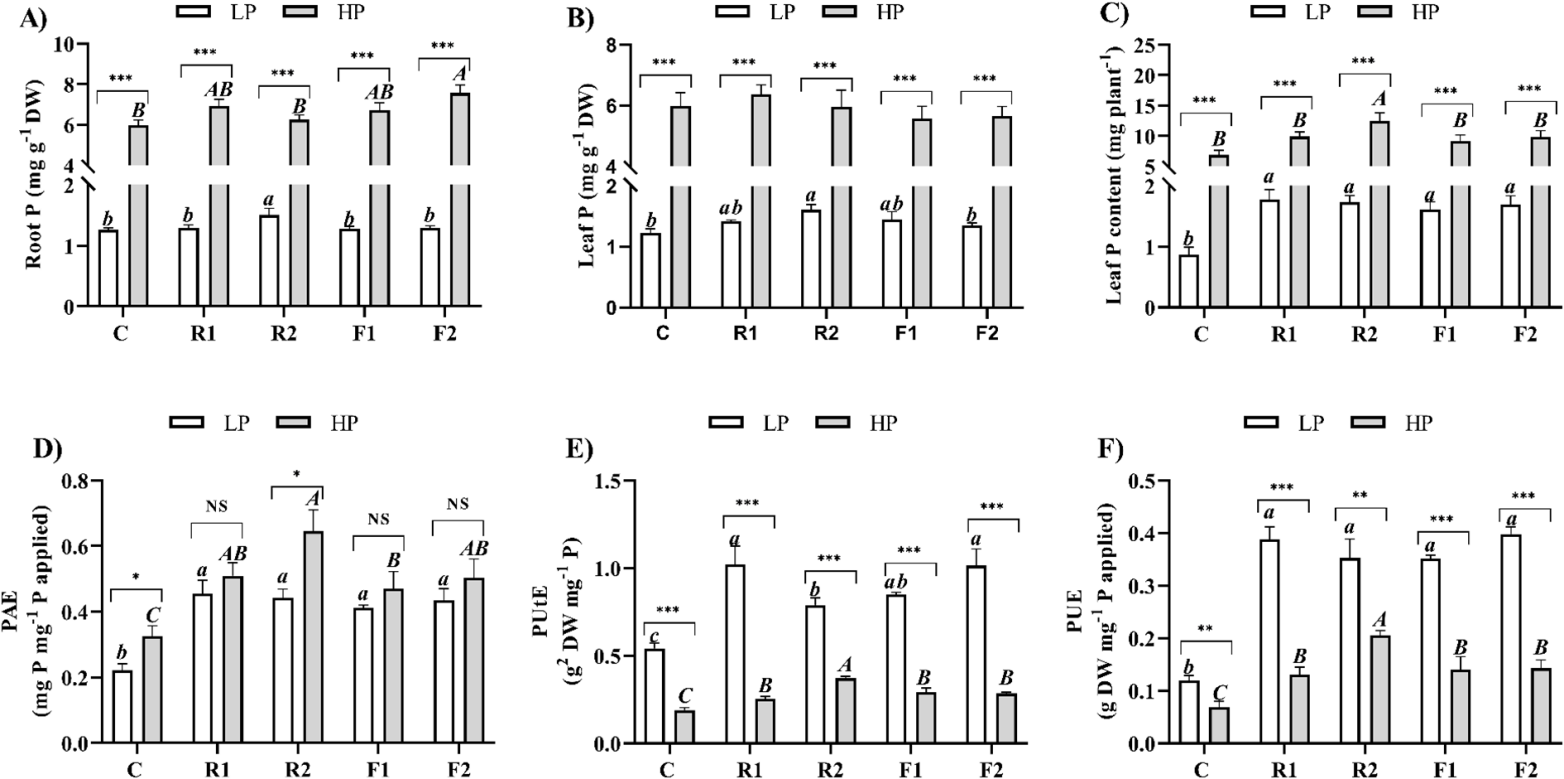
Root phosphorus (P) concentration **(A)**, leaf phosphorus (P) concentration **(B)** leaf phosphorus (P) content **(C)**, phosphorus acquisition efficiency (PAE) **(D)**, phosphorus utilization efficiency (PUtE) **(E)**, and phosphorus use efficiency (PUE) **(F)** in lettuce plants under low phosphorus (LP) and high phosphorus (HP) and treated with humic substances (HS) applied via root (R) at 0.40 mL L^-1^ (R1) and 0.60 mL L^-1^ (R2), and foliar (F) at 7.50 mL L^-1^ (F1) and 10.00 mL L^-1^ (F2), along with a control (C, HS-untreated). Values are means ± standard errors (n = 4). Different lowercase (LP) and uppercase (HP) letters indicate significant differences among HS treatments within each P level based on one-way ANOVA followed by Fisher’s least significant test (LSD, P< 0.05). Additionally, means values between LP and HP conditions within the same treatment were compared using a t-student test and differences were expressed as *(P< 0.05), **(P< 0.01), ***(P< 0.001), and NS (not significant, P > 0.05).

As expected, P concentration was higher in both roots and leaves of lettuce plants supplied with HP (**Figures 3A, B**). Consequently, leaf P content was also high in these plants (**Figure 3C**). Under P limitation, only the R2 treatment led to a significant rise in both root and leaf P concentrations (+20% and +31%, respectively) of plants compared to the respective control, although all HS treatments enhanced leaf P content. Under HP, root P concentration was significantly increased only by the F2 treatment (+26%) relative to the HP control, while leaf P concentration remained unaffected by HS applications; however, leaf P content was significantly increased by the R2 treatment (+82%).

With regard to P use efficiency indicators, PAE was higher in HS-untreated plants grown under HP conditions compared to LP, whereas the opposite trend was observed for PUtE and PUE (**Figures 3D–F**). All HS treatments significantly enhanced PAE, PUtE, and PUE compared to HS-untreated plants, regardless of the P supply level. While these improvements were remarkable and relatively similar in LP plants within each indicator across HS treatments, they were less pronounced under HP, with R2 treatment exhibiting the most notable effect.

### 3.3 Leaf acid phosphatase activity

**Figure 4:**
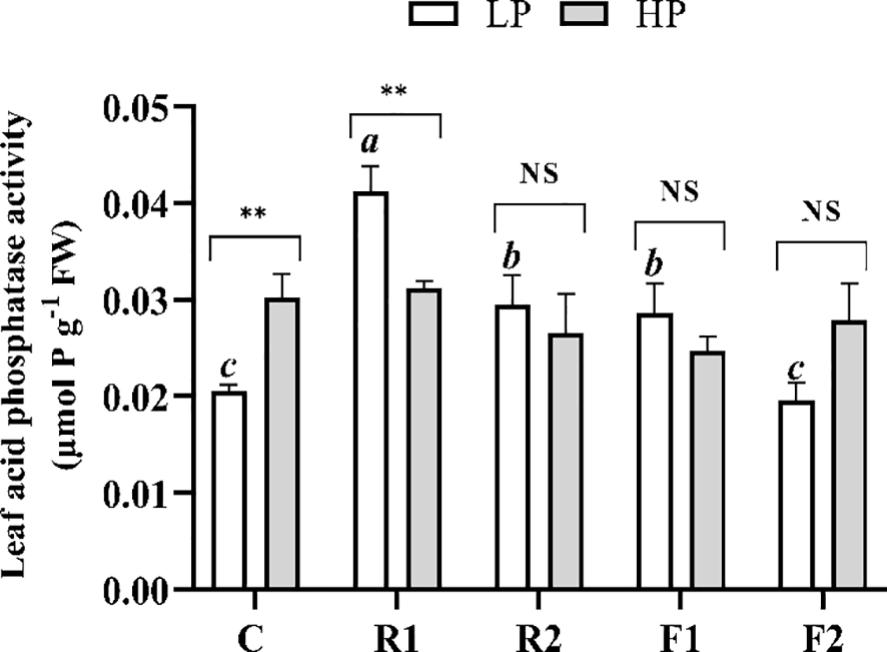
Leaf acid phosphatase activity in lettuce plants under low phosphorus (LP) and high phosphorus (HP) and treated with humic substances (HS) applied via root (R) at 0.40 mL L^-1^ (R1) and 0.60 mL L^-1^ (R2), and foliar (F) at 7.50 mL L^-1^ (F1) and 10.00 mL L^-1^ (F2), along with a control (C, non-HS). Values are means ± standard errors (n = 4). Different lowercase (LP) and uppercase (HP) letters indicate significant differences among HS treatments within each P level based on one-way ANOVA followed by Fisher’s least significant test (LSD, P< 0.05). Additionally, means values between LP and HP conditions within the same treatment were compared using a t-Student test and differences were expressed as **(P< 0.01) and NS (not significant, P > 0.05).

In HS-untreated plants, leaf acid phosphatase activity was notably higher under HP than under LP conditions (**Figure 4**). Under LP, treatments with R1, R2, and F1 significantly stimulated acid phosphatase activity compared to the untreated control, with R1 showing the greatest increase (+95%). In contrast, none of the HS treatments induced significant changes in acid phosphatase activity under HP conditions.

### 3.4 Chlorophyll content

In HS-untreated plants and those treated with F1, Chl *a*, Chl *b* and total chlorophyll concentrations were lower under P limitation compared to HP conditions (**Table 1**). The application of HS generally increased chlorophyll concentrations in both LP and HP plants relative to their respective untreated controls. Notably, treatments with R1, R2, and F2 under LP restored chlorophyll levels to values comparable to, or even exceeding, those measured in HP plants. The highest total chlorophyll accumulation was recorded in plants treated with R2 under LP and F1 under HP conditions.

**Table 1:** Chlorophyll (Chl) a, b, and total Chl content in lettuce under low phosphorus (LP), high phosphorus (HP), and humic substances (HS) applied via root (R) at 0.40 mL L^-1^ (R1) and 0.60 mL L^-1^ (R2), and foliar (F) at 7.50 mL L^-1^ (F1) and 10.00 mL L^-1^ (F2), along with a control (C, non-HS).

Treatments		Chl *a* *(mg 100 g^-1^ FW)*	Chl *b* *(mg 100 g^-1^ FW)*	Total Chl *(mg 100 g^-1^ FW)*
C	LP	14.77 ± 1.25*^d^*	3.86 ± 0.89*^d^*	18.63 ± 1.57*^d^*
	HP	20.36 ± 1.32^D*^	8.24 ± 1.15^C**^	28.59 ± 2.81^D*^
R1	LP	36.72 ± 1.31*^c^*	15.82 ± 0.95*^c^*	52.53 ± 2.49^c*^
	HP	25.62 ± 2.25^CD**^	14.22 ± 1.81*^B^*	39.84 ± 2.14*^CD^*
R2	LP	63.80 ± 1.81^a***^	38.73 ± 6.23^a***^	107.53 ± 7.41^a***^
	HP	33.73 ± 0.75*^BC^*	13.73 ± 1.13*^B^*	47.46 ± 3.49*^BC^*
F1	LP	49.40 ± 1.04*^b^*	27.64 ± 3.37*^b^*	77.05 ± 7.41*^b^*
	HP	69.00 ± 1.22^A**^	31.10 ± 3.24*^A^*	100.10 ± 8.75^A*^
F2	LP	47.06 ± 2.08*^b^*	29.49 ± 5.44^ab*^	76.55 ± 7.04^b*^
	HP	42.25 ± 1.29*^B^*	17.67 ± 1.61*^B^*	59.72 ± 3.72^D^

Values are means ± standard errors (n = 4). Different lowercase (LP) and uppercase (HP) letters indicate significant differences among HS treatments within each P level based on one-way ANOVA followed by Fisher’s least significant test (LSD, P**<** 0.05). Additionally, means values between LP and HP conditions within the same treatment were compared using a t-Student test and differences were expressed as *(P**<** 0.05), **(P**<** 0.01), ***(P**<** 0.001), and without asterisk (not significant, P > 0.05).

### 3.5 Glycine betaine accumulation

**Figure 5:**
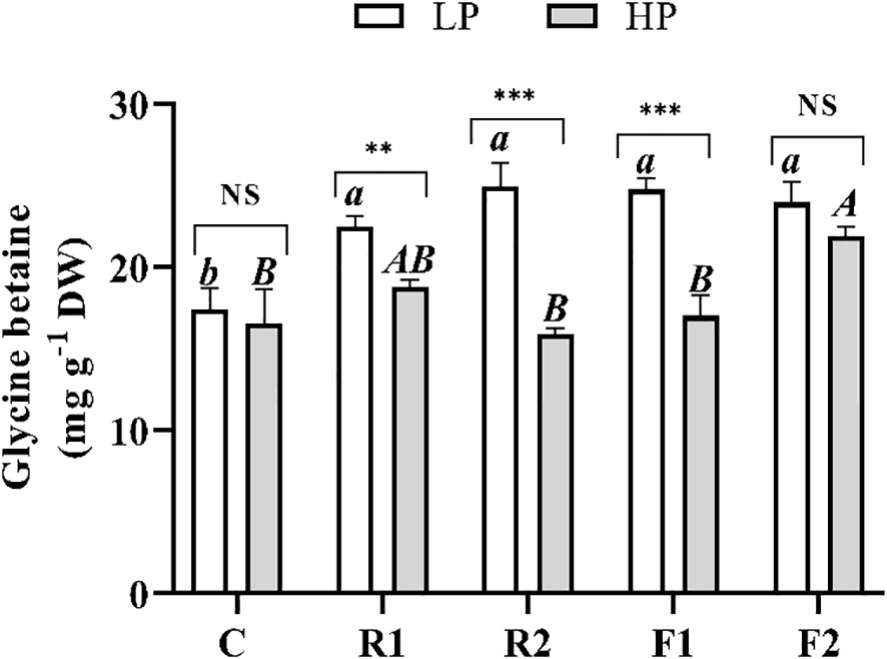
Leaf glycine betaine (GB) content in lettuce plants under low phosphorus (LP) and high phosphorus (HP) and treated with humic substances (HS) applied via root (R) at 0.40 mL L^-1^ (R1) and 0.60 mL L^-1^ (R2), and foliar (F) at 7.50 mL L^-1^ (F1) and 10.00 mL L^-1^ (F2), along with a control (C, non-HS). Values are means ± standard errors (n = 4). Different lowercase (LP) and uppercase (HP) letters indicate significant differences among HS treatments within each P level based on one-way ANOVA followed by Fisher’s least significant test (LSD, P< 0.05). Additionally, means values between LP and HP conditions within the same treatment were compared using a t-Student test and differences were expressed as **(P< 0.01), ***(P< 0.001), and NS (not significant, P > 0.05).

Glycine betaine accumulation was not affected by P supply in HS-untreated plants (**Figure 5**). However, treatments R1, R2, and F1 led to a significant increase in this metabolite under LP compared to HP conditions. While only the F2 treatment significantly promoted GB accumulation under HP, all HS treatments induced significant and comparable increases in GB under LP conditions.

### 3.6 Phenolic compound accumulation

**Figure 6:**
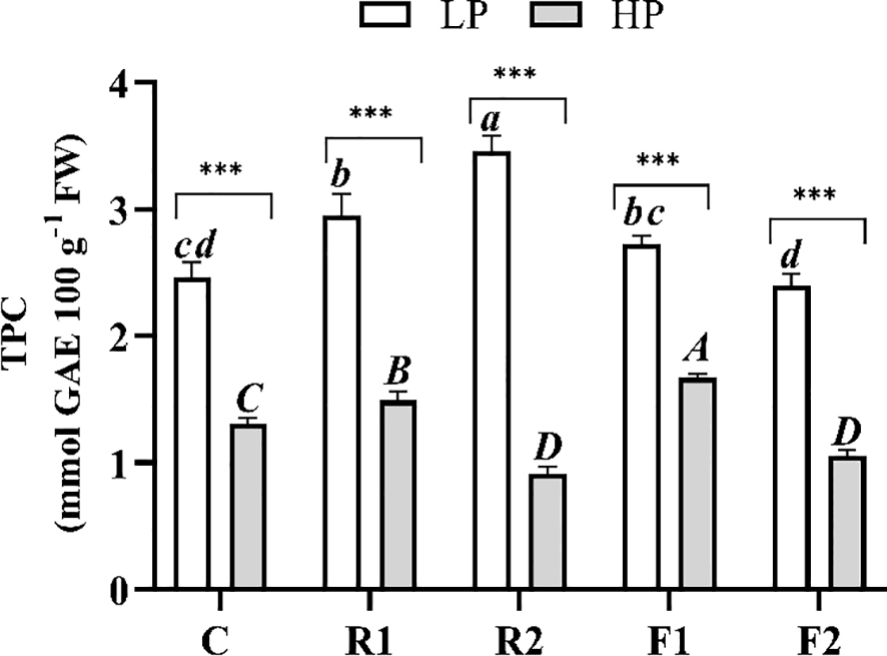
Leaf total phenolic content (TPC) in lettuce plants under low phosphorus (LP) and high phosphorus (HP) and treated with humic substances (HS) applied via root (R) at 0.40 mL L^-1^ (R1) and 0.60 mL L^-1^ (R2), and foliar (F) at 7.50 mL L^-1^ (F1) and 10.00 mL L^-1^ (F2), along with a control (C, non-HS). Values are means ± standard errors (n = 4). Different lowercase (LP) and uppercase (HP) letters indicate significant differences among HS treatments within each P level based on one-way ANOVA followed by Fisher’s least significant test (LSD, P< 0.05). Additionally, means values between LP and HP within each treatment were compared using a t-Student test and differences were expressed as **(P< 0.01), ***(P< 0.001), and NS (not significant, P > 0.05).

A significant higher total phenolic content (TPC) was observed in all plants grown under LP compared to HP, either treated or not with HS (**Figure 6**). Under P limitation, TPC was further enhanced by R1 and R2 applications (20% and 40%, respectively) compared to untreated plants, while F1 and F2 treatment had no significant effect. Under HP conditions, R1 and F1 treatments increased TPC (14% and 27%, respectively), whereas R2 and F2 treatment reduced TPC relative to the untreated control.

**Figure 7:**
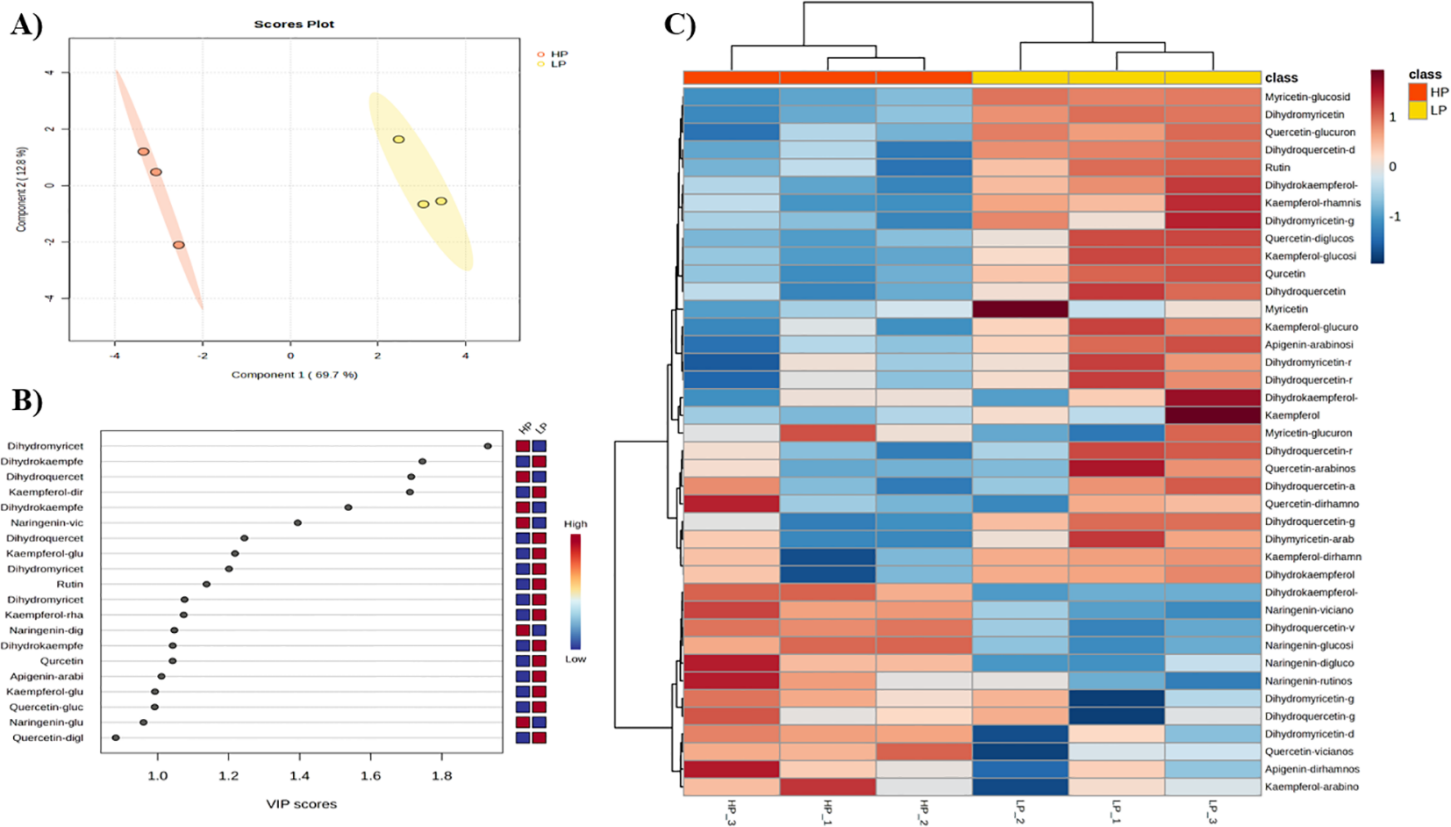
Metabolomic analysis showing the differences between humic substances (HS)-untreated plants under low phosphorus (LP) and high phosphorus (HP). **(A)** partial least squares-discriminant analysis (PLS-DA), highlighting the relationship between the predictor variables (X) and the categorical response (Y). The explained variance displayed in the plot corresponds to the portion of variability in X captured by the model. **(B)** variable importance in projection (VIP) scores and the weighted absolute regression coefficients. The colored boxes on the right represent the relative abundance of each corresponding metabolite across the different experimental groups. **(C)** heatmap visualization coupled with hierarchical clustering dendrogram, showing how the different metabolites are affected in both experimental groups. Each cell’s color on the map corresponds to a concentration value (higher values are represented in red, whereas lower values in blue).

**Figure 8:**
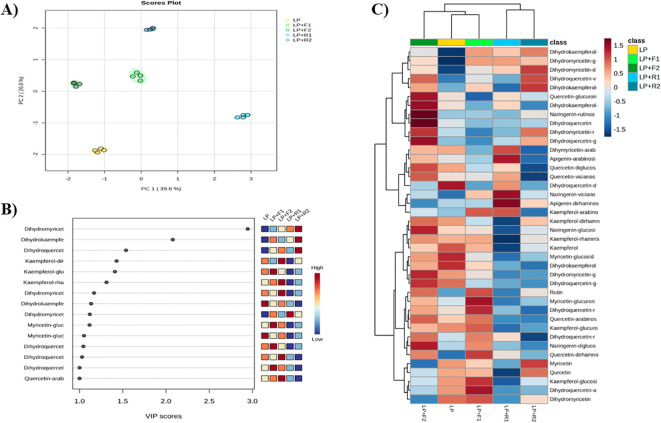
Metabolomic analysis showing the differences between treatments under low phosphorus (LP). Treatments are described as humic substances (HS) applied via root (R) at 0.40 mL L^-1^ (R1) and 0.60 mL L^-1^ (R2), and foliar (F) at 7.50 mL L^-1^ (F1) and 10.00 mL L^-1^ (F2), along with a control (C, non-HS). **(A)** partial least squares-discriminant analysis (PLS-DA), highlighting the relationship between the predictor variables (X) and the categorical response (Y). The explained variance displayed in the plot corresponds to the portion of variability in X captured by the model. **(B)** variable importance in projection (VIP) scores and the weighted absolute regression coefficients. The colored boxes on the right represent the relative abundance of each corresponding metabolite across the different experimental groups. **(C)** heatmap visualization coupled with hierarchical clustering dendrogram, showing how the different metabolites are affected in the different treatments. Each cell’s color on the map corresponds to a concentration value (higher values are represented in red, whereas lower values in blue).

**Figure 9:**
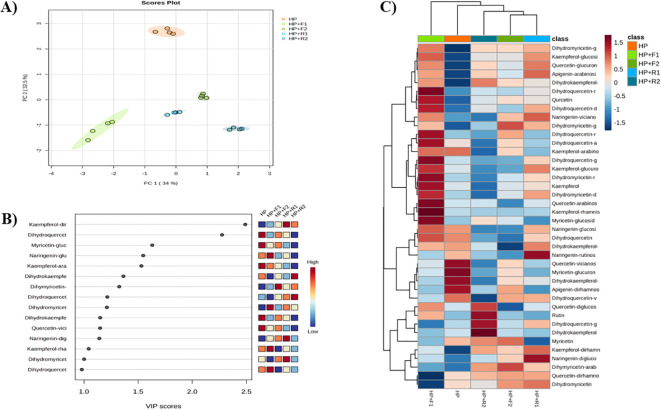
Metabolomic analysis showing the differences between treatments under high phosphorus (HP). Treatments are described as humic substances (HS) applied via root (R) at 0.40 mL L^-1^ (R1) and 0.60 mL L^-1^ (R2), and foliar (F) at 7.50 mL L^-1^ (F1) and 10.00 mL L^-1^ (F2), along with a control (C, non-HS). **(A)** partial least squares-discriminant analysis (PLS-DA), highlighting the relationship between the predictor variables (X) and the categorical response (Y). The explained variance displayed in the plot corresponds to the portion of variability in X captured by the model. **(B)** variable importance in projection (VIP) scores and the weighted absolute regression coefficients. The colored boxes on the right represent the relative abundance of each corresponding metabolite across the different experimental groups. **(C)** heatmap visualization coupled with hierarchical clustering dendrogram, showing how the different metabolites are affected in the different treatments. Each cell’s color on the map corresponds to a concentration value (higher values are represented in red, whereas lower values in blue).

In addition to TPC, the profile of phenolic compounds was determined in lettuce leaves to evaluate the effect of P supply (**Figure 7**) and HS treatments (**Figures 8**, **9**) on the accumulation of these secondary metabolites (**Supplementary Table 1**).

Partial Least Squares-Discriminant Analysis (PLS-DA) revealed a clear separation in metabolite accumulation between LP and HP conditions in HS-untreated plants, primarily along PC1 (**Figure 7A**). This clustering was mainly driven by 20 phenolic compounds listed in **Figure 7B**. Among them, dihydromyricetin, dihydrokaempferol, dihydroquercitin, and kaempferol-dirhamnoside showed the highest Variable Importance in Projection (VIP) values, indicating that these compounds were most influenced by P availability. Under LP conditions, a greater accumulation of these discriminant metabolites was observed compared to HP, suggesting a stress-related metabolic adjustment (**Figure 7B**). These major metabolites are part of 40 phenolic compounds detected in lettuce leaves, whose accumulation was generally more pronounced under LP, particularly kaempferol- and dihydrokaempferol-derivatives (**Figure 7C**).

The same analysis was performed to assess the effect of HS-treatments on the accumulation of specific phenolic compounds as a function of P supply and HS treatments (**Figures 8**, **9**). The PLS-DA analysis revealed clear discrimination among the different treatments applied to lettuce under LP conditions, suggesting that HS influenced the accumulation of specific phenolic compounds depending on the application method (root or foliar) and/or in a dose-dependent manner. Within the LP group of plants, those HS-untreated and F2-treated were separated from plants treated with R1 by PC1 scores, whereas PC2 allowed to differentiate R2-treated plants from both HS-untreated and R1-treated plants (**Figure 8A**). Dihydromyricetin and dihydrokaempferol were the key metabolites driving the discrimination observed under P limitation, showing higher accumulation in R2-treated plants and lower levels in untreated LP plants (**Figure 8B**). Considering the entire pool of phenolic compounds reported in **Figure 8C**, the F2 treatment was the most efficient to induce the highest accumulation of most metabolites compared to HS-untreated plants and those treated with R1, R2 and F1. Notable among these metabolites, the most accumulated were quercetin-glucuronide, dihydrokaempferol, naringenin-rutinoside, and dihydroquercetin.

Under HP conditions, PLS-DA analysis revealed that PC2 clearly distinguished HS-untreated plants from those treated with HS, while PC1 discriminated between F1-treated plants from those treated with F2 and R2 (**Figure 9A**). The phenolic compounds responsible for this separation between groups of plants were kaempferol-dirhamnoside and dihydroquercetin, which accumulated at higher levels in R1-treated plants and in the untreated control, respectively (**Figure 9B**). Consistent with the results observed under phosphorus limitation, HS treatments also influenced the concentration of phenolic metabolites under high phosphorus conditions. In this case, the F1 treatment induced the highest accumulation of phenolics, notably including dihydroquercetin-rhamnoside, dihydroquercetin-glucoside, dihydromyricetin-rhamnoside, kaempferol, quercetin-arabinoside, kaempferol-rhamnoside, and myricetin-glucoside, among others (**Figure 9C**).

### 3.7 Total antioxidant capacity

**Figure 10:**
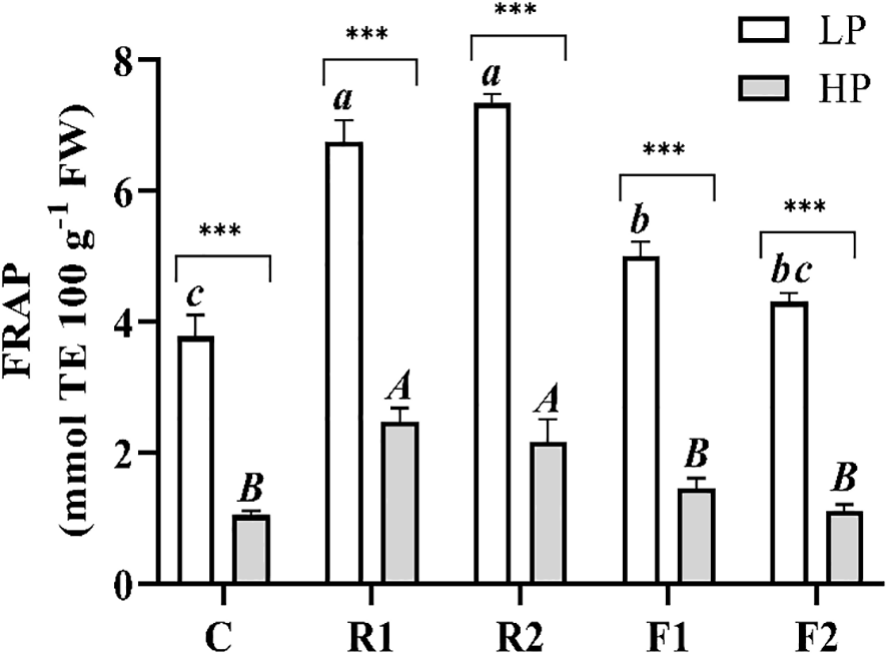
Leaf total antioxidant capacity measured through ferric reducing antioxidant power (FRAP) assay in lettuce plants under low phosphorus (LP) and high phosphorus (HP) and treated with humic substances (HS) applied via root (R) at 0.40 mL L^-1^ (R1) and 0.60 mL L^-1^ (R2), and foliar (F) at 7.50 mL L^-1^ (F1) and 10.00 mL L^-1^ (F2), along with a control (C, non-HS). Values are means ± standard errors (n = 4). Different lowercase (LP) and uppercase (HP) letters indicate significant differences among HS treatments within each P level based on one-way ANOVA followed by Fisher’s least significant test (LSD, P< 0.05). Additionally, means values between LP and HP within each treatment were compared using a t-student test and differences were expressed as ***(P<0.001).

Total antioxidant capacity, measured in lettuce leaves using the FRAP assay, was significantly higher under LP compared to HP conditions (**Figure 10**). Under LP conditions, the R1, R2, and F1 treatments significantly increased total antioxidant capacity by 78%, 94% and 32%, respectively, relative to the untreated control. Under HP conditions, R1 and R2 treatments also led to significant increases in FRAP values by 135% and 106%, respectively, relative to the HS-untreated control.

**Figure 11:**
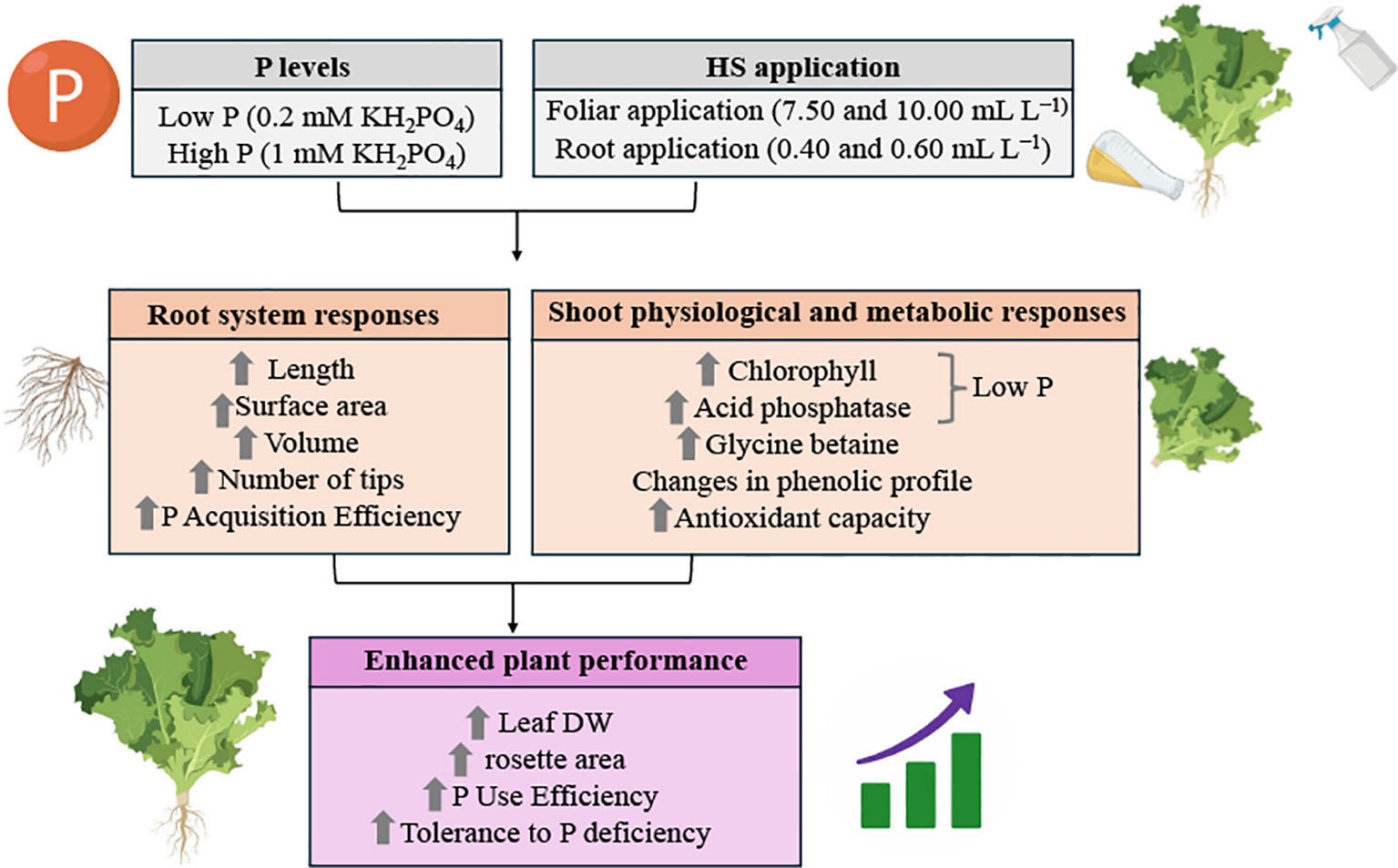
Proposed mechanisms of leonardite-derived humic substances in improving phosphorus use efficiency and plant tolerance to P deficiency.

The main physiological mechanisms of leonardite-derived HS underlying improvements in PUE and plant performance, as discussed above, are summarized in **Figure 11**.

## 4 Discussion

Fertilization is a widely used strategy to enhance crop yields under nutrient-limited conditions, with P deficiency being one of the most critical constraints (Meng et al., 2021). However, research has increasingly focused on biostimulants, such as HS, as a sustainable strategy to increase the uptake and utilization of nutrients supplied through conventional agronomic practices, aiming to reduce the overapplication of chemical fertilizers and their negative environmental impact (Raza et al., 2024).

In this study, a HS biostimulant derived from leonardite was applied to lettuce plants at varying doses and through different application methods, under both adequate (HP) and limited (LP) P supply conditions. The chemical composition of the HS used has been previously characterized by Barone et al. (2019), revealing C (51.1%) as the predominant element, followed by N (0.88%), and S (0.75%), with P accounting for only 0.06%. Based on this composition, the various physiological changes induced by HS application in lettuce were not attributable to their intrinsic P content, but rather to other chemical properties and biological activity of HS.

Reduced plant growth and productivity are among the main visible effects of P deficiency (Kaur et al., 2022; Kayoumu et al., 2023). Consistently, in our study we observed decreased lettuce growth under low P conditions. However, all HS treatments improved leaf DW compared to untreated plants under both P conditions. Our findings also indicate that the HS biostimulant effect was modulated by the plant nutrient status and was more pronounced when P was limiting (Jindo et al., 2020). In this case, the enhancement of lettuce growth was irrespective of the HS dose applied and mode of supplementation (root or foliar). Under HP conditions, the effect was instead more limited and appeared to depend on the application mode and dose of HS, with the high-dose root application (R2) being necessary to stimulate leaf growth. These results align with previous studies showing that HS can enhance lettuce yield under P-sufficient conditions (Guilayn et al., 2020; Atero-Calvo et al., 2023; Aguiar et al., 2024), and that HS-based products can alleviate P deficiency in crops (Santoro et al., 2023).

Regarding the effect of HS on root growth promotion, the lack of significant root biomass stimulation response under adequate P conditions may indicate that root growth was already optimized, thereby limiting the potential for additional enhancement by HS. Nevertheless, high-dose root application still improved certain root traits, including root length, surface area and tip number, indicating a comprehensive enhancement of the root system’s exploratory and absorptive capacity even in plants with adequate P supply. In particular, the increase in root length was likely driven by enhanced lateral root formation and branching, as reflected by the increase in root tip number, which collectively translated into a greater HS-induced foraging capacity. The increase in root length induced by HS was also observed by de Azevedo et al. (2019) in *Zea mays* L. This effect was attributed to enhanced root plasma membrane H^+^-ATPase activity, which facilitates H^+^ release into the apoplast, thereby activating expansin proteins and increasing cell wall plasticity, leading to greater cell elongation. Root growth stimulation is a key factor in enhancing P acquisition under P deficiency, as it enables greater soil exploration, which directly influences plant performance (Reddy et al., 2020). Our data reveal that application of the leonardite-derived HS significantly enhanced root growth under P-limitation. Notably, beyond increases in root biomass, distinct changes in root architecture were observed, depending on the specific HS treatment. Both root treatments and foliar application at the lower HS dose increased root length, promoting deeper root growth, while the F2 treatment enhanced root volume and diameter, thus favoring radial root expansion, higher root to shoot nutrient translocation efficiency, and greater storage capacity. Nunes et al. (2019) also reported increased length, diameter, and number of lateral roots in *Zea mays* L. treated with HA, attributing this growth to changes in energy metabolism proteins, such as 2-Cys peroxidase and glutathione proteins, which may also explain our results. Consequently, these architectural modifications likely contributed to improved P acquisition and homeostasis, supporting overall plant growth under P-deficient conditions (Nunes et al., 2019; Lynch et al., 2021).

The observed modifications of root architecture in HS-treated plants may have contributed to improved P acquisition efficiency (PAE) (Jing et al., 2022), particularly under P limitation, resulting in greater P uptake and content compared to untreated plants, which was consistent with findings in maize treated with two different lignohumates (Santoro et al., 2023). These effects may additionally be associated with increased expression and activity of root P transporters, as previously reported (Jindo et al., 2016). Notably, under P-limited conditions, all HS treatments increased total leaf P content, although only R2 treatment led to a rise in leaf P concentration. This likely occurred because leaf biomass increased more than P uptake, resulting in unchanged P concentration but higher total leaf P content due to the greater overall leaf biomass. Furthermore, the P absorbed and translocated to the leaves in HS-treated plants may have been more efficiently used for the synthesis of P organic compounds, as indicated by higher PUtE, remarkably high under P limitation, thereby contributing to enhanced plant growth (Zhang et al., 2020). These results are relevant as they indicate that HS application may improve internal P mobilization and utilization in lettuce plants (Veneklaas et al., 2012; Hasan et al., 2016). In support of this, PUE, defined as the amount of biomass produced per unit of P supplied (Navarro-León et al., 2022), was also enhanced by HS treatments under P-limited conditions. Consistent with our results, Hartina et al. (2025) observed that HA enhanced PAE and PUE in rice plants, supporting plant growth. Similar effects have also been reported in other crops, such as maize (Yuan et al., 2022) and wheat (Gao et al., 2023). Thus, our findings suggest that HS-induced improvements in PAE and PUE could contribute to enhanced lettuce growth under both P conditions.

Enhancement of PUE is commonly associated with the increased activities of enzymes such as acid phosphatase, which participates in the remobilization of Pi from intracellular orthophosphate monoesters in old leaves or vacuoles (Omoarelojie et al., 2025). The increase in acid phosphatase activity in lettuce leaves following HS root application and F1 treatment may have thus contributed to the improved PUtE and PUE observed under P shortage, similarly to results reported by Santoro et al. (2023) in maize. However, the effects of HS on leaf phosphatase activity and P remobilization remain largely unclear, as most of the existing literature focuses on HS-induced stimulation of phosphatase activity in the root or rhizosphere compartments (Yang et al., 2021; Yuan et al., 2022). Although most HS treatments stimulated leaf phosphatase activity under P limitation, we cannot rule out the possibility that HS also influenced the activity of this enzyme in the roots and rhizosphere, including other phosphorus-metabolizing enzymes such as phytase (Santoro et al., 2023).

The reduction in crop yields under P-limited conditions is associated with disruptions in several essential physiological processes, including photosynthesis (Kayoumu et al., 2023). Chlorophylls, the primary photosynthetic pigments responsible for light harvesting, are often diminished under P deficiency, further limiting the plant’s ability to capture light energy and affecting CO_2_ assimilation (Shu et al., 2023). This reduction may occur because several enzymes in the Chl biosynthetic pathway require P-containing cofactors or intermediates. Decreased Chl concentrations under P deficiency were observed in HS-untreated P limited plants, consistent with reports in *Citrus grandis* L. (Meng et al., 2021) and *L. sativa* L. (Cela et al., 2022).

While we did not assess photosynthetic activity directly, previous studies have shown that HS and other types of biostimulants can promote chlorophyll biosynthesis and accumulation, potentially enhancing photosynthetic capacity (Ertani et al., 2011; Atero-Calvo et al., 2024b; Velasco-Clares et al., 2024; Wang et al., 2024). In our study, applications of leonardite-derived HS increased Chl concentration, with this effect being more pronounced under LP compared to HP across most treatments. Similarly, Kaya et al. (2020) reported that sulfur-enriched leonardite and HA enhanced Chl concentration in maize under P deficiency, leading to improved photosynthesis and crop productivity. Lv et al. (2024) also observed increased total Chl content and dry biomass in maize treated with fulvic acids and under P deficiency. Considering the documented correlation between Chl concentration, photosynthetic efficiency, and crop yield (Yang et al., 2022), our findings support the role of HS in promoting Chl accumulation, which may contribute to improved plant growth under P limitation.

We additionally found that leonardite-derived HS helped plants to mitigate osmotic stress associated with P deficiency, consistent with previous reports on humic acids (Bayat et al., 2021). This effect appears to be mediated through enhanced synthesis and accumulation of compatible solutes. Among these, GB, a quaternary ammonium compound, supports plant stress tolerance through osmotic adjustments and osmoprotection (Hernandez-Leon and Valenzuela-Soto, 2022), stabilization of proteins and enzymes, antioxidant defense, protection of photosystem II, and prevention of Chl degradation (de Souza Júnior et al., 2022). While GB accumulation is well-documented under drought and salinity (De la Torre-González et al., 2018), its behavior under nutrient stress, including P shortage, is less explored (Oukaltouma et al., 2021). In our study, P-limited and P-sufficient plants showed similar GB levels when HS-untreated. However, HS application increased GB accumulation in P-limited plants. Oukaltouma et al. (2021) showed that increased GB levels contributed to maintaining cell membrane stability under combined water and P deficiencies, thereby sustaining plant performance. This finding supports our results, suggesting a positive role of GB in improving lettuce growth under P-limited conditions in plants treated with HS.

The total antioxidant capacity of lettuce plants was evaluated using the FRAP assay, which measures the ability of antioxidants to reduce Fe³^+^ to Fe²^+^. This assay primarily reflects the activity of non-enzymatic antioxidants such as glutathione, ascorbate, and phenolic compounds (Noperi-Mosqueda et al., 2020), which help mitigate oxidative stress caused by elevated levels of ROS, primarily resulting from disturbances to photosynthetic electron flux (Foyer and Hanke, 2022; Okla et al., 2024). The enhanced antioxidant activity of HS-treated plants was partly associated with changes in the profile and accumulation of phenolic compounds occurring in lettuce under different P supply. Some phenolics may have indeed more antioxidant potential than others. However, HS may also stimulate the biosynthesis of antioxidants other than phenolics, thereby contributing to overall antioxidant capacity as reported in tomato plants under heat stress (Khan et al., 2020) and in pepper under salinity conditions (Akladious and Mohamed, 2018) and in maize subjected to P shortage and treated with humates (Santoro et al., 2023). Such multifaceted antioxidant enhancement may explain the observed lack of a perfect correlation between total phenolic content and total antioxidant capacity. Notably, the HS treatments via root application were generally the most effective in promoting total antioxidant capacity compared to untreated plants, irrespective of P supply.

Focusing on phenolic compounds, they constitute a major group of antioxidants, synthesized *via* the phenylpropanoid pathway, which involves a sequence of enzymatic reactions starting from phenylalanine (Saini et al., 2024; Rao and Zheng, 2025). Phosphorus deficiency resulted in total phenolic accumulation to counter oxidative stress, as also found in different species such as *Zea mays* L (Shukla and Prithiviraj, 2021), *Artemisia argyi* L (Wang et al., 2023b), *Hevea brasiliensis* Müll. Arg (Saengwilai et al., 2023), and *L. sativa* L (Cela et al., 2022).

Additionally, phenolic compounds are associated with increased shelf life in lettuce, contributing to reduce browning, a common indicator of spoilage (Peng and Simko, 2023). The different treatments with leonardite-derived HS increased phenol accumulation in lettuce leaves. HS are known to stimulate the phenylpropanoid pathway by enhancing the activity of phenylalanine (tyrosine) ammonia-lyase (PAL/TAL) (Schiavon et al., 2010), a common target of biostimulants (Ertani et al., 2013, 2018). This effect may be attributed to the chemical characteristics of HS, including phenolic, alcoholic, and carboxylic functional groups, and by stimulation of C and N metabolism (Schiavon et al., 2010). Consistent with the characterization reported by Barone et al. (2019), the presence of aromatic-C functional groups (39.7%), along with the C and N content of the leonardite-derived HS, may have contributed to the phenylpropanoid pathway activation and, consequently, modulated phenolic accumulation. Moreover, the increase in Chl content under HS treatments could improve primary metabolism and sugar production, thereby supporting phenol biosynthesis (Conselvan et al., 2017). Our data also show that phenolic biosynthesis was differentially regulated by the mode and dose of HS application depending on P supply, with root applications being more effective in promoting total phenol accumulation under P shortage. We hypothesize that HS applied to the roots may have triggered a systemic signaling cascade that more effectively activated phenol biosynthesis in leaves than direct foliar application. This was perhaps due to the central role of roots in long-distance signaling, hormone modulation involved in phenylpropanoid pathway activation (Fragoso et al., 2014), and nutrient-driven metabolic shifts (Olaetxea et al., 2018; De Hita et al., 2020). In contrast, foliar application may have resulted in limited, localized effects due to potential cuticle absorption barriers or mild phytotoxicity (De Hita et al., 2020), reducing its effectiveness in triggering systemic phenolic production. In the present study we found that HS modulated the accumulation of specific phenolic compounds, mainly belonging to the flavonoid class (Gatti et al., 2024). In particular, foliar application of HS at the F2 dose resulted in the greatest increase in several metabolites under LP conditions, including quercetin-glucuronide, dihydrokaempferol, naringenin-rutinoside, and dihydroquercetin. The accumulation of these compounds may reflect a metabolic adjustment aimed at counteracting oxidative damage caused by P scarcity, as these metabolites contribute to ROS scavenging and protection of the photosynthetic apparatus (Singh et al., 2021; Jan et al., 2022; Arikan et al., 2023). In this context, Luo et al. (2019) reported increased levels of quercetin-3,4-O-di-β-glucopyranoside in sensitive maize seedlings under P-deficient conditions, establishing a link between Pi-responsive genes and the production of specific secondary metabolites associated with stress responses. The role of biostimulants in modulating the levels of specific phenolic compounds under abiotic stress has been previously reported. For instance, Ouhaddou et al. (2025) observed increased kaempferol levels in lettuce under drought stress following the application of compost amendments and beneficial microorganisms, which contributed to improved plant tolerance. Similarly, ascorbic acid application in basil increased quercetin levels and promoted plant growth under saline conditions (Yolcu and Yilmaz, 2025).

The observation that in HP plants treated with HS, the F1 treatment enhanced the accumulation of dihydroquercetin-rhamnoside, dihydroquercetin-glucoside, dihydromyricetin-rhamnoside, kaempferol, quercetin-arabinoside, kaempferol-rhamnoside, and myricetin-glucoside, supports the role of HS as signaling molecules, able to up-regulate plants defenses also when plants are not subjected to external stress, as well as priming agents against potential stresses. These compounds are indeed closely connected in the same metabolic network being also part of the flavonol/dihydroflavonol branch of the phenylpropanoid pathway and are among the most effective ROS scavengers in plants (Mahmud et al., 2023).

## 5 Conclusions

The application of leonardite-derived HS to lettuce effectively promoted overall growth, irrespective of the P supply. Although the positive effect of HS on root biomass was evident only under P limitation, improvements in root traits related to nutrient acquisition were also observed in plants grown with adequate P, especially following high-dose root applications. The growth responses were accompanied by increased PAE, PUtE, PUE and acid phosphatase activity, with stronger effects under P limitation. These results highlight the potential of leonardite-derived HS to promote both P acquisition and internal mobilization, allowing for increased leaf biomass. In addition to growth benefits, leonardite-derived HS also increased GB levels, total phenolic content and overall total antioxidant capacity, with these effects being more pronounced in P-limited plants, particularly when HS were applied to the roots. This underscores the major role of HS-root interactions for the triggering of metabolic processing occurring in leaves, apparently more efficient that HS-leaf interactions.

Our findings indicate that root and foliar HS applications may differentially influence phenolic metabolism under limited-P conditions. Root doses promoted a broader antioxidant response whereas foliar application may induce specific biosynthesis pathway stimulating the accumulation of certain flavonoids. Notably, HS treatments differentially affected the accumulation of individual phenolic compounds, with responses depending on both the application method and dose. These results suggest that HS may modulate plant secondary metabolism through distinct biochemical mechanisms, paving the way for future molecular studies to elucidate HS-mechanisms of action.

Overall, the leonardite-derived HS used in this study enhanced tolerance to P deficiency and enhanced growth and quality traits in lettuce under adequate P supply. Therefore, the results from this study support the agronomic potential of this biostimulant to reduce the reliance on chemical fertilizers, reducing their negative environmental impact and contributing to more sustainable crop production systems.

## Data Availability

The original contributions presented in the study are included in the article/**Supplementary Material**. Further inquiries can be directed to the corresponding author.
